# A network meta-analysis: evaluating the efficacy and safety of concurrent proton pump inhibitors and clopidogrel therapy in post-PCI patients

**DOI:** 10.3389/fcvm.2024.1385318

**Published:** 2024-07-24

**Authors:** Ming-Ying Ai, Yan-Zuo Chen, Chien-Liang Kuo, Wei-Lun Chang

**Affiliations:** Department of Pharmacy, Far Eastern Memorial Hospital, New Taipei, Taiwan

**Keywords:** major adverse cardiovascular events (MACEs), gastrointestinal (GI) bleeding, clopidogrel, proton pump inhibitors (PPIs), post-percutaneous coronary intervention (PCI)

## Abstract

**Introduction:**

The objective of this research was to evaluate the risk of major adverse cardiovascular events (MACEs) associated with the use of various proton pump inhibitors (PPIs) in combination with clopidogrel in patients who underwent percutaneous coronary intervention (PCI).

**Methods:**

To accomplish this, we analyzed data from randomized controlled trials and retrospective cohort studies sourced from key electronic databases. These studies specifically examined the effects of different PPIs, such as lansoprazole, esomeprazole, omeprazole, rabeprazole, and pantoprazole, when used in conjunction with clopidogrel on MACEs. The primary focus was on the differential impact of these PPIs, while the secondary focus was on the comparison of gastrointestinal (GI) bleeding events in groups receiving different PPIs with clopidogrel vs. a placebo group. This study's protocol was officially registered with INPLASY (INPLASY2024-2-0009).

**Results:**

We conducted a network meta-analysis involving 16 studies with a total of 145,999 patients. Our findings indicated that rabeprazole when combined with clopidogrel, had the lowest increase in MACE risk (effect size, 1.05, 95% CI: 0.66–1.66), while lansoprazole was associated with the highest risk increase (effect size, 1.48, 95% CI: 1.22–1.80). Esomeprazole (effect size, 1.28, 95% CI: 1.09–1.51), omeprazole (effect size, 1.23, 95% CI: 1.07–1.43), and pantoprazole (effect size, 1.38, 95% CI: 1.18–1.60) also significantly increased MACE risk. For the secondary outcome, esomeprazole (effect size, 0.30, 95% CI: 0.09–0.94), omeprazole (effect size, 0.34, 95% CI: 0.14–0.81), and pantoprazole (effect size, 0.33, 95% CI: 0.13–0.84) demonstrated an increased potential for GI bleeding prevention.

**Conclusions:**

In conclusion, the combination of lansoprazole and clopidogrel was found to significantly elevate the risk of MACEs without offering GI protection in post-PCI patients. This study is the first network meta-analysis to identify the most effective regimen for the concurrent use of clopidogrel with individual PPIs.

**Systematic Review Registration:**

https://inplasy.com/inplasy-2024-2-0009/, identifier (INPLASY2024-2-0009).

## 1 Introduction

Antiplatelet therapy is now the standard treatment for patients who have percutaneous coronary intervention (PCI). After a balloon injury and the insertion of a stent, the inner layer of the blood vessel at that location becomes damaged. This leads to the activation of both the coagulation cascade and platelets (1, 2). After PCI, a key focus is on preventing blood clots in the newly widened arteries, which is where medications such as clopidogrel come into play. Clopidogrel is an antiplatelet drug, essential in post-PCI management. It works by inhibiting platelets in the blood from clumping together to form clots. This action is particularly important in patients who have had stents placed during PCI, as stents increase the risk of clot formation within the artery (3).

Clopidogrel, usually taken in combination with aspirin, significantly reduces the risk of stent thrombosis, a serious complication where a blood clot forms on the stent, potentially leading to major cardiovascular events (MACEs) (4). The duration of clopidogrel therapy can vary based on the type of stent used and the patient's overall risk profile. Recent advancements in drug-eluting stents have influenced the recommended length of clopidogrel therapy (5). Continuous monitoring and follow-up are essential to manage any side effects of clopidogrel, such as bleeding risk, and to adjust treatment plans as needed for the individual patient. The goal is to balance the prevention of clotting with the risk of excessive bleeding, optimizing patient outcomes post-PCI (6).

To alleviate these side effects, PPIs such as esomeprazole, lansoprazole, pantoprazole, omeprazole, and rabeprazole have been introduced. PPIs are a class of medications widely used to treat conditions caused by excessive stomach acid production. They work by irreversibly blocking the hydrogen/potassium ATPase enzyme system of the gastric parietal cells, effectively reducing gastric acid secretion. PPIs are primarily used to manage gastroesophageal reflux disease (GERD), peptic ulcers, and Zollinger–Ellison syndrome (7). They are also prescribed to prevent and treat gastric ulcers induced by nonsteroidal anti-inflammatory drugs (NSAIDs) and to eradicate *Helicobacter pylori* infections in combination with antibiotics.

Combining PPIs with clopidogrel is beneficial for reducing the risk of GI bleeding in patients undergoing antiplatelet therapy. PPIs effectively decrease stomach acid, protecting the GI lining from damage, while clopidogrel prevents blood clots. The coadministration of antiplatelet agents and PPI is often a clinical choice for physical due to a decrease in the risk of GI bleeding (8). However, there are concerns about the potential interaction between clopidogrel and PPIs. The main concern is that both drugs use the same metabolic enzyme CYP2C19. PPIs might impede the conversion of clopidogrel into its active form through CYP2C19. This could result in lower blood levels of the active metabolite and possibly diminish clopidogrel's effectiveness in preventing platelet aggregation (9). It is noteworthy that different PPIs utilize different liver enzymes for metabolism. Due to the drug–drug interaction (DDI) concerns, the choice of PPI and clopidogrel should always be cautious. To figure out the effect of MACEs on coadministration in different PPIs with clopidogrel is important. Our study aims to determine which specific PPIs, when coadministered with clopidogrel, might have the lowest MACEs and GI bleeding risk.

## 2 Materials and methods

We executed this study following the guidelines outlined in the Preferred Reporting Items for Systematic Reviews and Meta-Analysis (PRISMA) extension for network meta-analysis (PRISMA NMA) (10). The study has been registered on INPLASY under the registration number INPLASY2024-2-0009 (11). The approval from the ethical review board or securing informed consent from participants was unnecessary.

### 2.1 Database searches and study identification

Two authors (M-YA and W-LC) independently conducted electronic searches across the following databases, including PubMed, Cochrane Reviews, Cochrane Central, Web of Science, and ClinicalTrials.gov. The search utilized the following keywords: (“Clopidogrel”) AND (“Percutaneous coronary intervention”) AND (“Lansoprazole” OR “Omeprazole” OR “Esomeprazole” OR “Pantoprazole” OR “Rabeprazole” OR “Dexlansoprazole” OR “Proton pump inhibitors”). The systematic review and network meta-analysis search spanned from the earliest entry in each database up to the most recent search date (24 January 2024). First, the two authors systematically assessed the titles and abstracts of identified studies for eligibility through a consensus process. In instances where consensus could not be reached by the initial two reviewers, a third author was consulted. There were no language restrictions imposed on this search.

### 2.2 Inclusion and exclusion criteria

The network meta-analysis was structured according to the PICO (population, intervention, comparison, and outcome), encompassing the following criteria: (1) P, human participants with post-PCI using clopidogrel; (2) I, coadministrated PPI; (3) C, placebo group without intervention; and (4) O, MACE risk. The study applied the subsequent inclusion criteria: (1) the randomized controlled trials and retrospective cohort studies that enrolled patients after PCI using clopidogrel with or without individual PPIs; (2) the randomized controlled trials and retrospective studies that enrolled patients after PCI using clopidogrel with or without individual PPIs longer than a month; (3) the placebo group receiving no PPIs; and (4) studies providing available data on MACEs.

The exclusion criteria applied in this review and network meta-analysis comprised the following: (1) patients without PCI intervention, (2) studies lacking individual PPI analysis of MACEs risk, (3) studies without antiplatelet agent clopidogrel, (4) studies without placebo/control groups, (5) data that are either incomplete or not accessible despite efforts to reach the authors through email, and (6) research involving participants who were also part of a trial previously incorporated into our analysis.

### 2.3 Modeling for network meta-analysis

While performing our network meta-analysis, we followed certain guidelines in building our model to ensure uniformity and minimize variability. Our paired comparisons were restricted to either clopidogrel plus PPI vs. placebo or control group. We deliberately excluded comparisons between clopidogrel plus PPI and other antiplatelet agents combined with PPI, such as prasugrel. This choice was based on the understanding that incorporating other antiplatelet agents could create diverse network structures because of the distinct characteristics of these treatments, potentially leading to inconsistent results (12).

### 2.4 Methodological quality appraisal

For assessing the methodological soundness of the studies included in our analysis, we used the Cochrane risk of bias tool for randomized control trials (version 2, RoB 2, based in London, UK) (13) and Newcastle-Ottawa scale (NOS) assessment for non-randomized control studies (14). The Cochrane risk of bias tool instrument evaluates six crucial aspects to determine the quality of a study, including the process of randomization, compliance with the intervention, management of missing outcome data, measurement of outcomes, selective outcome reporting, and the overall likelihood of bias. The NOS comprises three domains, namely, selection of study groups, comparability of study groups, and assessment of outcome to determine the quality of a study.

### 2.5 Primary outcome

In our study, the primary outcome focused on assessing the effect size of various PPIs on the incidence of MACEs, when coadministered with clopidogrel. We analyzed lansoprazole, esomeprazole, omeprazole, pantoprazole, and rabeprazole, comparing their impact on MACEs without PPI usage.

### 2.6 Secondary outcomes

In our study, the secondary outcome focused on evaluating the impact of individual PPIs coadministered with clopidogrel on the risk of GI bleeding. This analysis aimed to understand the size effect of each PPI had on reducing GI bleeding risks in comparison to a placebo group. The specific PPIs examined were esomeprazole, omeprazole, pantoprazole, rabeprazole, and lansoprazole.

### 2.7 Data extraction, management, and conversion

The data extraction was independently carried out by two authors, M-YA and W-LC. This process involved collecting various types of information from the studies, such as demographic details, study design, specifics of the PPI, and both primary and secondary outcomes. The procedures for extracting data, converting, and merging the results were performed in line with the guidelines provided in the Cochrane Handbook for Systematic Reviews of Interventions, as well as other pertinent medical literature (15–19).

### 2.8 Statistical analyses

Given the diversity of the included studies in our article, we employed a random-effects model for the network meta-analysis. The analysis was executed using the MetaInsight software (version 4.0.2, Complex Reviews Support Unit, National Institute for Health Research, London, UK), operating within a frequentist statistical framework. MetaInsight, a web-based tool for network meta-analysis, utilizes the netmeta package in R software to perform frequentist statistical analyses.

In the initial stage of our analysis, we created forest plots and network plots to visually represent the pairwise comparisons from each study. Additionally, we generated forest plots to assess the relative risk of MACEs and GI bleeding compared to a placebo group. These plots offered a detailed view of the outcomes, showing effect sizes as point estimates accompanied by 95% confidence intervals (95% CI). We ranked the different types of PPIs based on their efficacy and presented numerical data for both direct and indirect comparisons in tables. To check for any inconsistencies in the data, we carried out inconsistency tests. The threshold for statistical significance was set at a two-tailed *p*-value of <0.05.

### 2.9 Sensitivity analyze

To improve the trustworthiness of our study's results, we employed the one-study-removed sensitivity analysis method, which involves sequentially excluding each study. This approach helped ensure that the effect estimates from any individual study did not disproportionately influence the overall findings. By systematically removing each study one by one from the analysis of MACEs and GI bleeding, we were able to determine whether the ultimate conclusions and the rankings of the studies remained stable.

### 2.10 Publication bias

We evaluated possible publication bias in accordance with the procedures outlined in the Cochrane Handbook for Systematic Reviews of Interventions (15). To illustrate this, we generated a funnel plot using the Comprehensive Meta-Analysis Software, version 4 (BioStat, Englewood, NJ, USA), specifically examining the comparisons with the placebo group. Additionally, Egger's regression test was utilized to quantitatively ascertain the extent and significance of any potential publication bias.

## 3 Results

### 3.1 Study identification and network model formation

**Figure 1:**
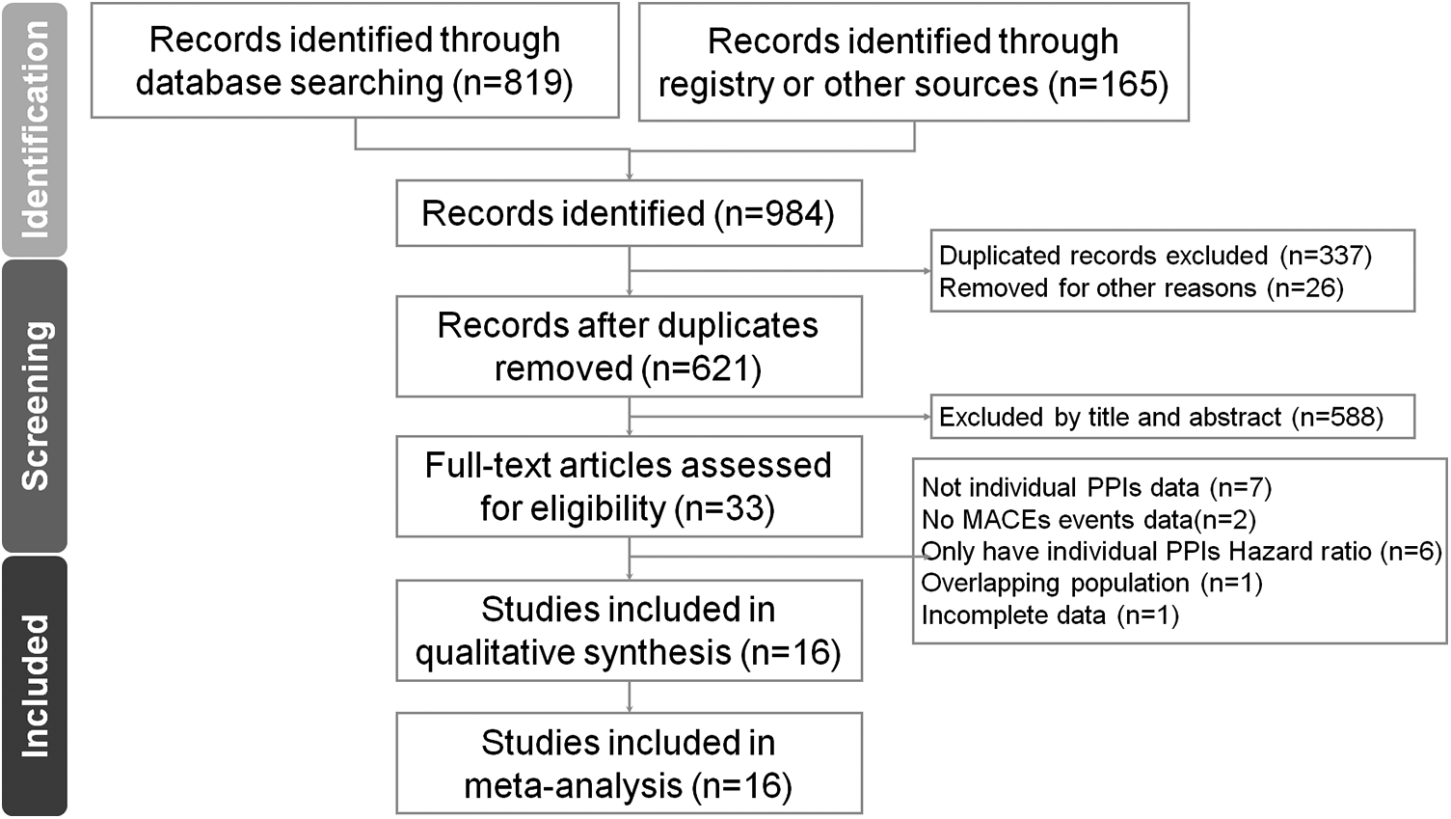
PRISMA flowchart.

Figure 1 displays the PRISMA flowchart illustrating the process of our literature search. The checklist for the PRISMA NMA extension can be found in Supplementary Table S1. Our selection process involved filtering out duplicate articles and discarding those irrelevant to our research, based on screening of their titles and abstracts, which resulted in the inclusion of 16 studies (20–35). These were either randomized controlled trials or retrospective or prospective cohort studies.

**Figure 2:**
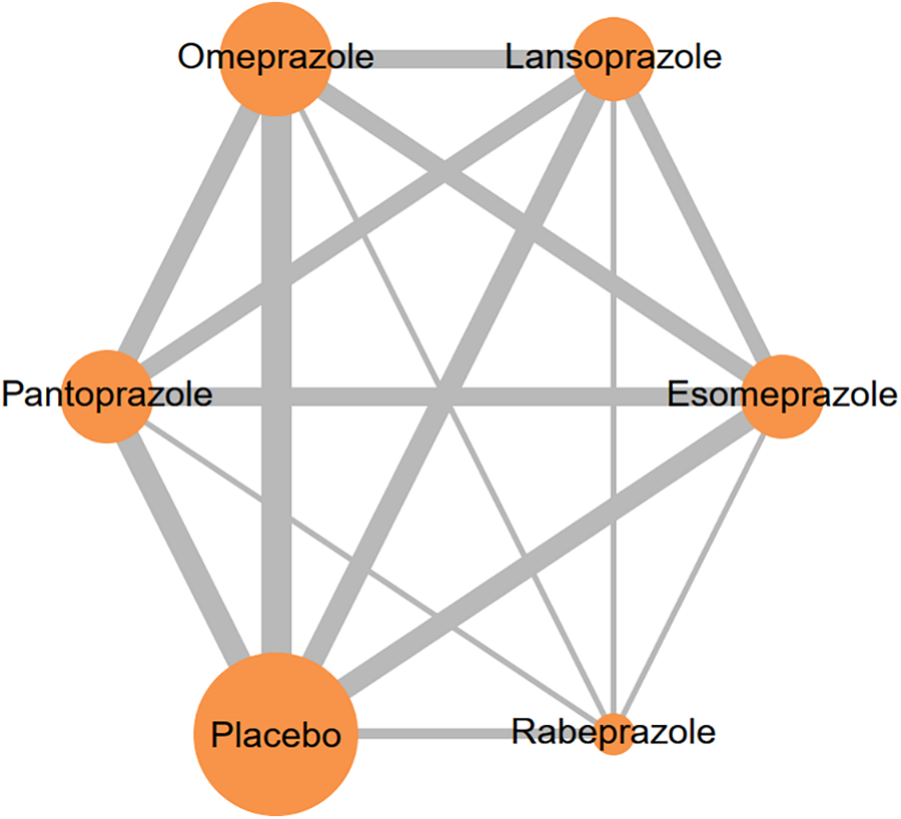
Network plots illustrate the effects of different PPIs concurrent with clopidogrel on MACE risk. The size of each node and thickness of each line represent the number of trials included in the analysis.

In total, our analysis encompassed 16 studies involving 145,999 individuals. The proton pump inhibitors (PPIs) evaluated in these studies included lansoprazole, esomeprazole, omeprazole, pantoprazole, and rabeprazole. The network model illustrating the interaction with clopidogrel co-treatment with individual PPI is shown in Figure 2. For additional information regarding the inclusion criteria and details about the studies, please refer to Table 1.

**Table 1:** Summary of the included studies in concurrent PPIs and clopidogrel resulted in MACEs and GI bleeding.

Study (year)	Designs	Clinical condition	Follow-up (months)	Country	PPI type	PPIs used (No. of patients)		Placebo (No. of patients)		PPI used (No. of patients)		Placebo (No. of patients)	
						MACEs	No MACEs	MACEs	NoMACEs	GI bleeding	No GI bleeding	GI bleeding	No GI bleeding
Bhatt et al. (20)	RCT	Mixed	3.5	Multinational	O	55	1,876	54	1,885	15	1,876	53	1,885
Yano et al. (21)	RCT	ACS	12	Japan	O	8	65	11	65	–	–	–	–
Ng et al. (22)	RCT	ACS	4.5	Hong Kong	E	7	163	5	148	1	163	9	148
Zhang et al. (23)	RCT	ACS	6	China	L	7	53	5	51	–	–	–	–
Wei et al. (24)	RCT	ACS	6	China	P	48	118	33	80	2	123	13^a^	84
Ren et al. (25)	RCT	ACS	1	China	O	22	86	22	86	0	86	2	86
Gaglia et al. (26)	Non-RCT	Mixed	12	USA	O, P, E, L, R	O, 8; P, 2; E, 28; L, 4; R, 2	O, 41; P, 35; E, 185; L, 41; R, 16	40	502	–	–	–	–
Ray et al. (27)	Non-RCT	Mixed	12	USA	O, P, E, L, R	O, 41; P, 272; E, 30; L, 91; R, 9	O, 660; P, 4,349; E, 690; L, 1,042; R, 275	580	13,001	O:5, P:34, E:5, L:14, R:1	O, 704; P, 4,629; E, 747; L, 1,096; R, 288	117	9,621
Kreutz et al. (28)	Non-RCT	Mixed	12	USA	O, P, E, L	O, 579; P, 484; E, 811; L, 191	O, 2,307; P, 1,653; E, 3,257; L, 785	1,766	9,862	–	–	–	–
Rossini et al. (29)	Non-RCT	Mixed	12	Italy	O, P, L	O, 5; P, 14; L, 67	O, 125; P, 178; L, 855	8	170	–	–	–	–
Simon et al. (30)	Non-RCT	ACS	12	France	O,P,E,L	O, 43; P, 12; E, 20; L, 1	O, 627; P, 78; E, 258; L, 37	60	679	–	–	–	–
Hokimoto et al. (31)	Non-RCT	Mixed	18	Japan	R	9	103	13	188	–	–	–	–
Yasu et al. (32)	Non-RCT	Mixed	12	Japan	R	5	50	6	124	4	103	16	199
Macaione et al. (33)	Non-RCT	ACS	12–18	Italy	O, P, E, L	O, 22; P, 3; E, 8; L, 5	O, 52; P, 42; E, 14; L, 13	7	55	–	–	–	–
Abukhalil et al. (34)	Non-RCT	ACS	12	Palestine	O, P, E, L	O, 2; P, 18; E, 23; L, 2	O, 11; P, 113 E, 198; L, 9	14	112	–	–	–	–
Maret-Ouda et al. (35)	Non-RCT	Mixed	12	Swedish	O, P, E	O, 1;126; P, 187; E, 157	O, 26,789; P, 4,138; E, 3,540	2,134	64,064	–	–	–	–

RCT, randomized controlled trial; non-RCT, non-randomized controlled trial; O, Omeprazole; P, pantoprazole; E, esomeprazole; L, Lansoprazole; R, rabeprazole; ACS, acute coronary syndrome; MACEs, major adverse cardiovascular events; GI, gastrointestinal; PPIs, proton pump inhibitors.

^a^ Identified patients with symptoms such as vomiting; brown, red, or black gastric juice; bloody stool; and black stool with a positive result on an occult blood test.

### 3.2 Methodological quality of the included studies

The evaluation of the methodological quality of the studies is shown in Supplementary Table S2–S3 and Figure S1. The studies identified with a potential bias risk demonstrated protocol variations among different study groups, potentially influencing adherence and the results of the interventions. Comprehensive information on the risk of bias evaluation is available in Supplementary Table S2–S3.

### 3.3 Primary outcome: MACE risk after receiving individual PPI

**Figure 3:**
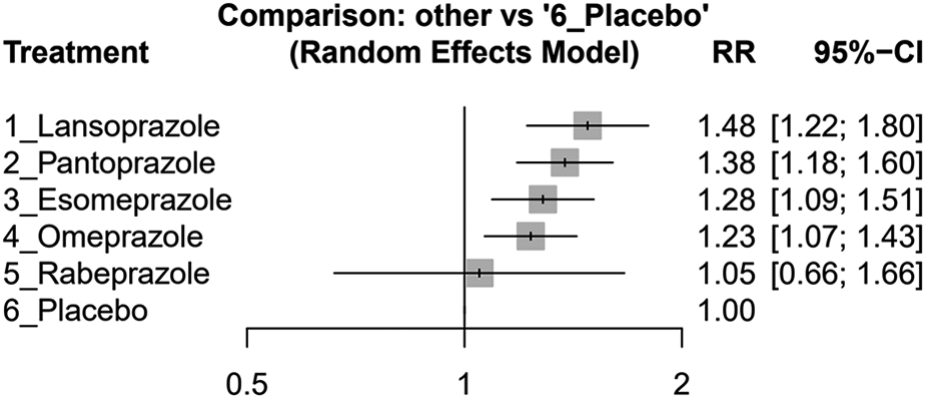
Forest plots illustrating the risk ratio (RR) of MACEs in clopidogrel concurrent with different PPIs and placebo groups among post-PCI patients.

In our study, lansoprazole (effect size, 1.48, 95% CI: 1.22–1.80), esomeprazole (effect size, 1.28, 95% CI: 1.09–1.51), omeprazole (effect size, 1.23, 95% CI: 1.07–1.43), and pantoprazole (effect size, 1.38, 95% CI: 1.18–1.60) showed significant increasing MACEs compared without PPI usage. However, rabeprazole (effect size, 1.05, 95% CI: 0.66–1.66) did not show a significant difference compared with the placebo group (see Figure 3). Please see Supplementary Figure S2 for a comprehensive overview of the direct comparisons between different study groups, as detailed in each study.

We have also analyzed the individual outcomes and total mortality. Total mortality included four studies, myocardial infarction included eight studies, stent thrombosis included three studies, stroke included two studies, and cardiac death included four studies. The results are shown in Supplementary Figure S3. Our analysis revealed no significant differences in total mortality, stroke, or cardiac death between individual PPIs combined with clopidogrel. However, esomeprazole (effect size, 4.41, 95% CI: 1.63–11.92) and omeprazole (effect size, 3.7, 95% CI: 1.39–9.87) were found to significantly increase the incidence of stent thrombosis. Additionally, these two PPIs were also found to significantly increase the risk of myocardial infarction (esomeprazole (effect size, 1.59, 95% CI: 1.07–2.34) and omeprazole (effect size, 1.41, 95% CI: 1.00–1.97).

The individual PPI coadministrated with clopidogrel influenced the MACE risk on their effect size, as shown in Table 2. The result indicated that rabeprazole with clopidogrel had a similar risk to MACEs in the group with clopidogrel without any PPI. The lansoprazole group showed the highest risk of MACEs, followed by esomeprazole, omeprazole, and pantoprazole. The detailed comparison and ranking are shown in Table 2.

**Table 2:** Pairwise comparison and ranking the risk of MACEs in different PPIs concurrent with clopidogrel.

Lansoprazole	1.08 (0.87, 1.35)	1.11 (0.87, 1.43)	1.07 (0.84, 1.36)	2.16 (1.11, 4.23)	1.59 (1.29, 1.97)
1.08 (0.88, 1.32)	Pantoprazole	1.14 (0.94, 1.38)	1.10 (0.91, 1.32)	1.60 (0.82, 3.12)	1.38 (1.18, 1.61)
1.15 (0.93, 1.43)	1.07 (0.90, 1.28)	Esomeprazole	0.99 (0.82, 1.19)	1.30 (0.65, 2.58)	1.32 (1.11, 1.57)
1.20 (0.98, 1.47)	1.12 (0.94, 1.32)	1.04 (0.87, 1.24)	Omeprazole	1.82 (0.92, 3.60)	1.25 (1.08, 1.46)
1.41 (0.87, 2.30)	1.31 (0.82, 2.12)	1.22 (0.76, 1.98)	1.18 (0.73, 1.90)	Rabeprazole	1.11 (0.69, 1.77)
1.48 (1.22, 1.80)	1.38 (1.18, 1.60)	1.28 (1.09, 1.51)	1.23 (1.07, 1.43)	1.05 (0.66, 1.66)	Placebo

### 3.4 Secondary outcome: Gi bleeding risk after receiving individual PPI

**Figure 4:**
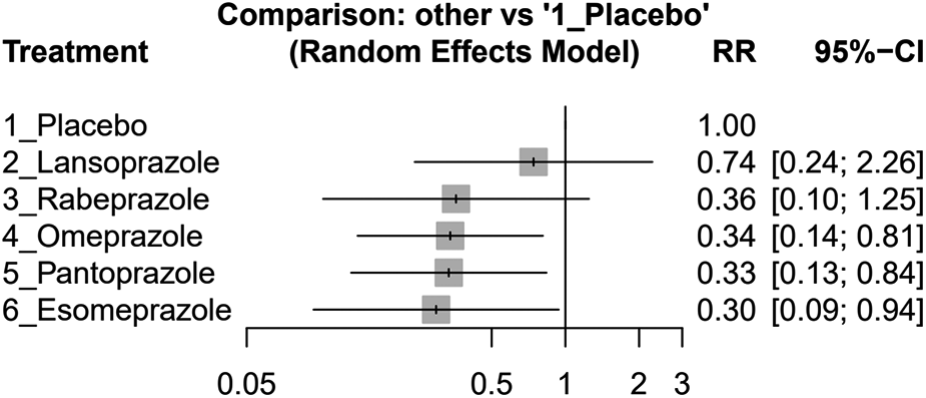
Forest plots of the risk ratio (RR) of GI bleeding in clopidogrel concurrent with different PPIs and placebo groups among post-PCI patients.

The individual PPI coadministrated with clopidogrel influenced the GI bleeding risk on their size, as shown in Figure 4. The result indicated that esomeprazole (effect size, 0.30, 95% CI: 0.09–0.94), omeprazole (effect size, 0.34, 95% CI: 0.14–0.81), and pantoprazole (effect size, 0.33, 95% CI: 0.13–0.84) significantly reduced the GI bleeding risk compared with placebo. However, rabeprazole (effect size, 0.36, 95% CI: 0.10–1.25) and lansoprazole (effect size, 0.74, 95% CI: 0.24–2.26) did not show significant difference compared with the placebo group. The detailed comparison and ranking are shown in Supplementary Table S4.

### 3.5 Inconsistency test

The construction of the network involved the establishment of nodes and the execution of both direct and indirect comparisons to assess consistency. Supplementary Table S5 presents the outcomes of the coadministrated clopidogrel with individual PPI evaluations, and Supplementary Table S6 contains the findings related to GI bleeding risk. With all conducted comparisons yielding *p*-values >0.05, there was no indication of inconsistency between the direct and indirect comparisons.

### 3.6 Sensitivity analyses

The one-study removal analysis yielded consistent rankings and maintained the clinical importance of each type of PPI. Lansoprazole, pantoprazole, and esomeprazole were found to significantly increase the incidence of MACEs, while omeprazole remained at borderline significance. Rabeprazole was unique in showing no significant impact on the rise in MACE risk, as illustrated in Supplementary Figure S5a–p. These findings affirm the robustness of our study's results, demonstrating that they remain stable regardless of whether individual studies are included or excluded, and are not affected by adjustments to the assumed values in the calculations.

### 3.7 Publication bias

Refer to Supplementary Figure S6 to view the funnel plot. The results from Egger's test, with a *p*-value of 0.302, suggest the absence of significant publication bias.

## 4 Discussion

Our study results showed that the coadministration of esomeprazole, lansoprazole, pantoprazole, and omeprazole with clopidogrel significantly increased the risk of MACEs compared to using clopidogrel alone. However, when rabeprazole was coadministered with clopidogrel, there was no significant difference in MACEs compared to the placebo group. In the secondary analysis, the combination of esomeprazole, omeprazole, and pantoprazole with clopidogrel significantly reduced the risk of GI bleeding compared to clopidogrel alone. In contrast, the combinations of lansoprazole and rabeprazole with clopidogrel did not show a significant reduction in GI bleeding risk compared to the placebo group.

Clopidogrel, commonly combined with aspirin, has been a standard treatment post-PCI. Prior research has indicated that clopidogrel, when used as a post-PCI medication, is effective in preventing cardiovascular (CV) events, death, or thrombosis. However, the use of dual antiplatelet therapy or prolonged antiplatelet treatment significantly increases the risk of GI bleeding (36–38). To address this, PPIs have been recommended as a co-treatment with clopidogrel to reduce GI bleeding risk (39). However, it is widely known that clopidogrel and PPIs are both metabolized by the same liver enzyme, resulting in a significant DDI. Clopidogrel is a prodrug that initially needs to be metabolized by liver enzymes CYP1A2, CYP2C19, and CYP2B6 to form the intermediate compound 2-oxo-clopidogrel. In the second metabolic phase, 2-oxo-clopidogrel is further processed by enzymes CYP3A4/5, CYP2C9, CYP2C19, and CYP2B6 to become its active form, inhibiting platelet aggregation (40). The CYP2C19 plays a critical role in both phases of forming the active compound of clopidogrel, and then PPIs inhibit the CYP2C19 enzyme. Unlike clopidogrel, prasugrel requires only one hepatic CYP450-dependent metabolism step to convert to its active metabolite, involving CYP2B6, CYP2C9, CYP2C19, CYP2D6, and CYP3A4. Therefore, its anti-aggregation effect is less likely to be affected by CYP2C19 inhibitors. Ticagrelor binds to the ADP receptor at a different site than ADP, acting as an allosteric antagonist. It inhibits ADP-induced P2Y12 receptor signaling non-competitively and does not require metabolic activation to produce its active metabolite (41). A previous *in vivo* RCT study also demonstrated that for patients with AMI undergoing primary PCI, omeprazole reduced the incidence of gastrointestinal bleeding without diminishing ticagrelor's antiplatelet aggregation effect or increasing the risk of MACEs (42).

The extent of interaction between specific PPIs and clopidogrel remains a topic of debate (43). Our study aims to use network meta-analysis to identify the specific PPI least associated with MACEs and also had GI bleeding prevention.

PPIs are mainly metabolized by CYP2C19 and can inhibit this enzyme. The degree and duration of CYP2C19 inhibition vary among individual PPIs (44). Studies have shown distinct mechanisms among different PPIs. Lansoprazole and pantoprazole, being direct-acting inhibitors, can directly inhibit CYP2C19 and have a short elimination half-life, possibly having a less significant impact on clinical MACE risk. However, lansoprazole has been shown to strongly inhibit CYP2C19 *in vitro*, leading to controversy in findings. Omeprazole and esomeprazole irreversibly inhibit CYP2C19 at low concentrations through NADPH coenzyme assistance, known as metabolism-dependent inhibitors (MDIs) (45). Both of them were thought to be strong CYP2C19 inhibitors to interfere with clopidogrel’s turn to active form. Rabeprazole, which is believed to be metabolized not only through CYP enzyme-mediated pathways but also via non-enzymatic routes, is considered to have a lesser impact on clopidogrel (46). Ilaprazole, a new PPI, is believed to have a limited effect on the pharmacodynamics of clopidogrel, suggesting it may not be clinically relevant according to *in vitro* studies (47). Our study observed that only the combination of rabeprazole and clopidogrel did not significantly increase the risk of MACEs compared to placebo. In contrast, omeprazole, esomeprazole, pantoprazole, and lansoprazole all showed a significant increase in MACE risk. Notably, the combination of lansoprazole and clopidogrel was associated with the highest increase in MACE risk.

In recent years, attention has turned to how genetic differences can influence drug metabolism. Clopidogrel is a notable example, with its metabolism varying across races. Studies have revealed significant racial differences in clopidogrel metabolism. Genetic mutations, such as the single nucleotide variations in CYP2C19*2 and CYP2C19*3 compared to the CYP2C19*1, result in poor metabolism of clopidogrel due to reduced enzyme activity (48). Asian populations (12%–100%) more frequently exhibit these poor metabolizers (PM) of CYP2C19 genotypes compared to Caucasians and Africans (3%–5%) (49). Patients with these mutations have significantly reduced clopidogrel antiplatelet efficacy and a higher risk of CV events than those with normal genotypes (50). Interestingly, while there have been studies on CYP2C19 variations across races, there is a few of research on the pharmacokinetics and pharmacodynamics of clopidogrel–PPI co-metabolism across different racial groups and the effect of different CYP2C19 genotype impacted (51, 52). Recent meta-analyses found no significantly increased MACEs in groups without CYP2C19 variant alleles when any PPI was coadministrated with clopidogrel. In contrast, those with CYP2C19 variant alleles showed a significant increase in MACEs (53). To summarize, while some studies recommend PPIs with clopidogrel, especially for high bleeding risk populations, this may be controversial for Asian populations with a higher prevalence of variant alleles, potentially leading to more frequent MACEs.

Clopidogrel is a crucial drug used for patients post-PCI treatment. While clopidogrel reduces the risk of MACEs, it carries the potential side effect of GI bleeding, with patients at high risk of GI bleeding requiring careful consideration as guideline recommendations (9). Long-term use of antiplatelet agents can notably increase the risk of GI bleeding, particularly with dual antiplatelet therapy. In patients with a history of prior GI bleeding, *Helicobacter pylori* infection, or concurrent use of other drugs such as antiplatelets, anticoagulants, corticosteroids, or NSAIDs, the risk of GI bleeding may be further heightened. For these populations, combining PPIs with antiplatelet agents could be a strategy to mitigate GI bleeding risk (9). In our analysis of secondary outcomes, it was observed that lansoprazole and rabeprazole, when coadministered with clopidogrel, did not significantly enhance GI bleeding prevention compared to the group receiving clopidogrel alone. On the other hand, omeprazole, esomeprazole, and pantoprazole demonstrated a significant reduction in GI bleeding incidents compared to the placebo group. The esomeprazole combined with clopidogrel could reduce the most GI bleeding risk.

Another factor we should though about is the influence of the PPI dosage. Although our study and previous studies had shown that omeprazole and esomeprazole coadministrated with clopidogrel increased MACEs, studies by Yano et al. (omeprazole 10 mg) and Ng et al. (esomeprazole 20 mg) suggested that lower doses of PPIs might not increase the risk of MACEs while still providing bleeding protection. In our study, although the combination of esomeprazole or omeprazole with clopidogrel appeared to increase the risk of MACEs, both PPIs significantly reduced GI bleeding. Future research should aim to determine whether lower doses of omeprazole and esomeprazole can effectively prevent GI bleeding without increasing the risk of MACEs.

Our study has some limitations. Firstly, our primary objective was to assess the risk of MACEs, but there was variability in the definition of MACEs across the studies we analyzed. While most included myocardial infarction (MI), stroke, and/or cardiovascular death, some also considered other conditions (such as thrombosis). Secondly, the duration of follow-up in our studies varied widely, ranging from a minimum of one month to several years. Different follow-up durations post-PCI can indicate varying levels of MACE risk. Additional limitations included as follows: (1) The included studies varied in patient characteristics, such as a history of previous GI bleeding, *Helicobacter pylori* infection, and so on, which could increase bleeding risk when using clopidogrel. (2) The absence of data on the CYP2C19 genotypes. There is a need for further randomized, high-quality studies in this area.

## 5 Conclusions

In our network meta-analysis, we discovered that the combination of lansoprazole, pantoprazole, esomeprazole, and omeprazole with clopidogrel might increase the risk of major adverse cardiac events (MACEs). However, coadministration of rabeprazole with clopidogrel did not appear to significantly impact MACE risk. We also noted that using omeprazole, esomeprazole, and pantoprazole concurrently with clopidogrel could significantly reduce the risk of gastrointestinal (GI) bleeding compared to using clopidogrel alone. However, using rabeprazole and lansoprazole did not reduce GI bleeding risk in post-PCI patients. Lansoprazole combined with clopidogrel significantly increased MACEs and without GI bleeding protection function in post-PCI patients. Our study was the first network meta-analysis study to figure out the best regiment when concurrent clopidogrel with individual PPI. However, further randomized and high-quality studies are necessary to substantiate this conclusion.

## Data Availability

The original contributions presented in the study are included in the article/Supplementary Material, further inquiries can be directed to the corresponding author.
