# Supplementary material for: A network meta-analysis: evaluating the efficacy and safety of concurrent proton pump inhibitors and clopidogrel therapy in post-PCI patients
Source: Front Cardiovasc Med. 2024 Jul 24;11:1385318. doi: 10.3389/fcvm.2024.1385318 (PMC11303300; doi:10.3389/fcvm.2024.1385318)

Table S1 - PRISMA for network meta-analysis checklist

Section and Topic	#	Checklist item	Location
**Title**			
Title	1	Identify the report as a systematic review incorporating a network meta-analysis (or related form of meta-analysis).	Title
**Abstract**			
Structured summary	2	Provide a structured summary including, as applicable: **Background**: main objectives / **Methods**: data sources; study eligibility criteria, participants, and interventions; study appraisal; and synthesis methods, such as network meta-analysis. / **Results**: number of studies and participants identified; summary estimates with corresponding confidence/credible intervals; treatment rankings may also be discussed. Authors may choose to summarize pairwise comparisons against a chosen treatment included in their analyses for brevity. / **Discussion/conclusions**: limitations; conclusions and implications of findings. / **Other**: primary source of funding; systematic review registration number with registry name.	Abstract
**Introduction**			
Rationale	3	Describe the rationale for the review in the context of what is already known, including mention of why a network meta-analysis has been conducted.	Introduction
Objectives	4	Provide an explicit statement of questions being addressed, with reference to participants, interventions, comparisons, outcomes, and study design (PICOS).	Introduction
**Methods**			
Protocol & registration	5	Indicate whether a review protocol exists and where it can be accessed; and, if available, provide registration information, including registration number.	Materials and Methods
Eligibility criteria	6	Specify study characteristics (e.g., PICOS, length of follow-up) and report characteristics (e.g., years considered, language, publication status) used as criteria for eligibility, giving rationale. Clearly describe eligible treatments included in the treatment network, and note whether any have been clustered or merged into the same node (with justification).	Materials and Methods
Information sources	7	Describe all information sources (e.g., databases with dates of coverage, contact with study authors) in the search and date last searched.	Materials and Methods
Search	8	Present full electronic search strategy for at least one database, including any limits used, such that it could be repeated.	Materials and Methods
Study selection	9	State the process for selecting studies (i.e., screening, eligibility, included in systematic review, and, if applicable, included in the meta-analysis).	Materials and Methods
Data collection	10	Describe method of data extraction from reports (e.g., piloted forms, independently, in duplicate) and any processes for obtaining and confirming data.	Materials and Methods
Data items	11	List and define all variables for which data were sought (e.g., PICOS, funding sources) and any assumptions and simplifications made.	Materials and Methods
Network geometry	S1	Describe methods used to explore the geometry of the treatment network under study and potential biases related to it. This should include how the evidence base has been graphically summarized for presentation, and what characteristics were compiled and used to describe the evidence base to readers.	Materials and Methods
Risk of bias within	12	Describe methods used for assessing risk of bias of individual studies (including specification of whether this was done at the study or outcome level), and how this information is to be used in any data synthesis.	Materials and Methods
Summary measures	13	State the principal summary measures (e.g., risk ratio, difference in means). Also describe the use of additional summary measures assessed, such as treatment rankings, as well as modified approaches used to present summary findings from meta-analyses.	Materials and Methods

Planned methods of analysis	14	Describe the methods of handling data and combining results of studies for each network meta-analysis. This should include, but not be limited to: Handling of multi-arm trials; Selection of variance structure; Selection of prior distributions in Bayesian analyses; and Assessment of model fit.	Materials and Methods
Assessment of inconsistency	S2	Describe the statistical methods used to evaluate the agreement of direct and indirect evidence in the treatment network(s) studied. Describe efforts taken to address its presence when found.	Materials and Methods
Risk of bias across	15	Specify any assessment of risk of bias that may affect the cumulative evidence.	Materials and Methods
Additional analyses	16	Describe methods of additional analyses if done, indicating which were pre-specified. This may include, but not be limited to, the following: Sensitivity or subgroup analyses; Meta-regression analyses; Alternative formulations of the treatment network; and Use of alternative prior distributions for Bayesian analyses (if applicable).	Materials and Methods
**Results**			
Study selection	17	Give numbers of studies screened, assessed for eligibility, and included in the review, with reasons for exclusions at each stage, ideally with a flow diagram.	Results/ Figure 1
Network structure	S3	Provide a network graph of the included studies to enable visualization of the geometry of the treatment network.	Figure 2
Network geometry	S4	Provide a brief overview of characteristics of the treatment network. This may include commentary on the abundance of trials and randomized patients for the different interventions and pairwise comparisons in the network, gaps of evidence in the treatment network, and potential biases reflected by the network structure.	Results / Network model formation / Figure 2
Study characteristics	18	For each study, present characteristics for which data were extracted (e.g., study size, PICOS, follow-up period) and provide the citations.	Table 1
Risk of bias within	19	Present data on risk of bias of each study and, if available, any outcome level assessment.	Table S2-3, Figure S1
Results of individual studies	20	For all outcomes considered (benefits or harms), present, for each study: (1) simple summary data for each intervention group, and (2) effect estimates and confidence intervals. Modified approaches may be needed to deal with information from larger networks.	Table 1
Synthesis of results	21	Present results of each meta-analysis done, including confidence/credible intervals. In larger networks, authors may focus on comparisons versus a particular comparator (e.g. placebo or standard care), with full findings presented in an appendix. League tables and forest plots may be considered to summarize pairwise comparisons. If additional summary measures were explored (such as treatment rankings), these should also be presented.	Figure 3, Figure 4, Figure S2-3
Exploration for inconsistency	S5	Describe results from investigations of inconsistency. This may include such information as measures of model fit to compare consistency and inconsistency models, P values from statistical tests, or summary of inconsistency estimates from different parts of the treatment network.	Table S5, Table S6
Risk of bias across	22	Present results of any assessment of risk of bias across studies for the evidence base being studied.	Figure S5
Additional analyses	23	Give results of additional analyses, if done (e.g., sensitivity or subgroup analyses, meta-regression analyses, alternative network geometries studied, alternative choice of prior distributions for Bayesian analyses, and so forth).	Figure S4
**Discussion**			
Summary of evidence	24	Summarize the main findings, including the strength of evidence for each main outcome; consider their relevance to key groups.	Discussion
Limitations	25	Discuss limitations at study and outcome level (e.g., risk of bias), and at review level (e.g., incomplete retrieval of identified research, reporting bias). Comment on the validity of the assumptions, such as transitivity and consistency. Comment on any concerns regarding network geometry (e.g., avoidance of certain comparisons).	Discussion
Conclusions	26	Provide a general interpretation of the results in the context of other evidence, and implications for future research.	Conclusion
**Funding**			

Funding	27	Describe sources of funding for the systematic review and other support (e.g., supply of data); role of funders for the systematic review. This should also include information regarding whether funding has been received from manufacturers of treatments in the network and/or whether some of the authors are content experts with professional conflicts of interest that could affect use of treatments in the network.	Funding

PICOS, population, intervention, comparators, outcomes, study design.

Table S2 - Comprehensive evaluation of the quality of included studies was conducted using the Cochrane Risk of Bias 2 tool.

First author & Year	Randomization process	Intervention adherence	Missing outcome data	Outcome measurement	Selective reporting	Overall RoB
Bhatt 2010	L	L	L	L	L	L
Ren 2011	L	S[1]	L	L	L	S
Ng 2012	L	L	L	L	L	L
Yano 2012	L	S[2]	L	L	L	S
Zhang 2015	L	S[3]	L	L	L	S
Wei 2016	L	L	L	L	L	L

[1] The studies didn't provide allocation concealment details.
[2] The study was open-label study.
[3] The study was nonblind study.
H, high risk of bias; L, low risk of bias; S, some risk of bias.

Table S3 - Comprehensive evaluation of the quality of included studies was conducted using the Newcastle Ottawa Scale (NOS).

First Author	S[1]1	S2	S3	S4	C[2]1	E[3]1	E2	E3	Total
Gaglia 2009	*	*	*		*	*	*	*	7
Kreutz 2010	*	*	*	*	**	*	*	*	9
Ray 2010	*		*	*	*	*	*	*	7
Takeo 2010	*	*	*	*	*	*	*	*	8
Rossini 2011	*	*	*	*	*	*	*	*	8
Simon 2011	*	*	*	*	**	*	*	*	9
Francesca 2012	*	*	*	*	*	*	*	*	8
Hokimoto 2014	*	*	*	*	*	*	*	*	8
Maret-Ouda 2022	*		*	*	*	*	*	*	7
Abdalla 2023	*		*	*	*	*	*	*	7

[1] The Selection part assessment.
[2] The Comparability part assessment.
[3] The Outcome part assessment.

Table S4 - Pairwise comparison and ranking the risk of GI bleeding in different PPIs concurrent with clopidogrel.

**Placebo**	0.95 [0.28, 3.20]	2.44 [0.69, 8.60]	2.66 [1.10, 6.46]	2.72 [1.03, 7.17]	2.86 [0.86, 9.48]
1.35 [0.44, 4.12]	**Lansoprazole**	3.68 [0.37, 36.50]	1.80 [0.41, 7.93]	1.74 [0.50, 6.04]	1.91 [0.43, 8.41]
2.80 [0.80, 9.76]	2.07 [0.42, 10.20]	**Rabeprazole**	0.49 [0.04, 5.39]	0.47 [0.05, 4.53]	0.52 [0.05, 5.72]
2.95 [1.24, 7.05]	2.19 [0.61, 7.81]	1.06 [0.24, 4.64]	**Omeprazole**	0.97 [0.23, 4.04]	1.06 [0.21, 5.48]
2.99 [1.20, 7.49]	2.22 [0.67, 7.37]	1.07 [0.24, 4.69]	1.01 [0.33, 3.15]	**Pantoprazole**	1.10 [0.26, 4.58]
3.37 [1.06, 10.67]	2.50 [0.62, 10.09]	1.21 [0.23, 6.19]	1.14 [0.30, 4.33]	1.13 [0.31, 4.07]	**Esomeprazole**

Table S5 - Inconsistency test results of the risk ratio (RR) of MACEs in clopidogrel concurrent with different PPIs and placebo groups among post-PCI patients.

Comparison	Studies	NMA	Direct	Indirect	Difference	95CIL	95CIU	*p*-value
Esomeprazole:Lansoprazole	6	-0.14	-0.11	-0.24	0.13	-0.35	0.62	0.59
Esomeprazole:Omeprazole	7	0.04	-0.01	0.50	-0.51	-1.09	0.07	0.08
Esomeprazole:Pantoprazole	7	-0.07	-0.13	0.34	-0.47	-1.01	0.07	0.09
Esomeprazole:Placebo	8	0.25	0.28	-0.04	0.31	-0.25	0.88	0.28
Esomeprazole:Rabeprazole	2	0.20	0.26	0.15	0.11	-0.85	1.08	0.82
Lansoprazole:Omeprazole	7	0.18	0.07	0.52	-0.45	-0.92	0.03	0.06
Lansoprazole:Pantoprazole	7	0.07	0.08	0.04	0.03	-0.51	0.58	0.90
Lansoprazole:Placebo	8	0.39	0.46	0.06	0.41	-0.10	0.92	0.11
Lansoprazole:Rabeprazole	2	0.35	0.77	-0.14	0.91	-0.07	1.89	0.07
Omeprazole:Pantoprazole	8	-0.11	-0.10	-0.18	0.08	-0.38	0.54	0.72
Omeprazole:Placebo	11	0.21	0.22	-0.02	0.24	-0.41	0.89	0.46
Omeprazole:Rabeprazole	2	0.16	0.60	-0.26	0.85	-0.10	1.81	0.08
Pantoprazole:Placebo	9	0.32	0.32	0.33	-0.01	-0.63	0.61	0.98
Pantoprazole:Rabeprazole	2	0.27	0.47	0.08	0.39	-0.56	1.34	0.42
Rabeprazole:Placebo	4	0.05	0.10	-1.95	2.05	-0.81	4.91	0.16

Table S6 - Inconsistency test results for the risk ratio (RR) of GI bleeding in clopidogrel concurrent with different PPIs and placebo groups among post-PCI patients.

Comparison	Studies	NMA	Direct	Indirect	Difference	95CIL	95CIU	*p*-value
Esomeprazole:Lansoprazole	1	-0.92	-0.65	-2.99	2.34	-2.03	6.71	0.29
Esomeprazole:Omeprazole	1	-0.13	-0.06	-0.28	0.22	-2.60	3.03	0.88
Esomeprazole:Pantoprazole	1	-0.12	-0.09	-0.23	0.14	-3.13	3.41	0.93
Esomeprazole:Placebo	2	-1.22	-1.05	-3.21	2.16	-2.18	6.50	0.33
Esomeprazole:Rabeprazole	1	-0.19	0.66	-0.92	1.58	-1.70	4.85	0.35
Lansoprazole:Omeprazole	1	0.78	0.59	1.33	-0.74	-3.63	2.14	0.61
Lansoprazole:Pantoprazole	1	0.80	0.55	4.01	-3.46	-8.15	1.23	0.15
Lansoprazole:Placebo	1	-0.30	0.05	-2.25	2.29	-0.82	5.41	0.15
Lansoprazole:Rabeprazole	1	0.73	1.30	0.19	1.11	-2.08	4.30	0.50
Omeprazole:Pantoprazole	1	0.01	-0.03	0.09	-0.13	-2.47	2.22	0.92
Omeprazole:Placebo	3	-1.08	-0.98	-4.09	3.12	-1.74	7.98	0.21
Omeprazole:Rabeprazole	1	-0.05	0.72	-0.53	1.24	-1.81	4.29	0.42
Pantoprazole:Placebo	2	-1.10	-1.00	-1.93	0.93	-2.10	3.95	0.55
Pantoprazole:Rabeprazole	1	-0.07	0.75	-0.68	1.43	-1.56	4.41	0.35
Rabeprazole:Placebo	2	-1.03	-0.89	-11.43	10.53	-0.58	21.65	0.06

Figure S1 - Summary of quality assessment for the randomized control trials included in the current network meta-analysis using version 2 of the Cochrane risk-of-bias tool.

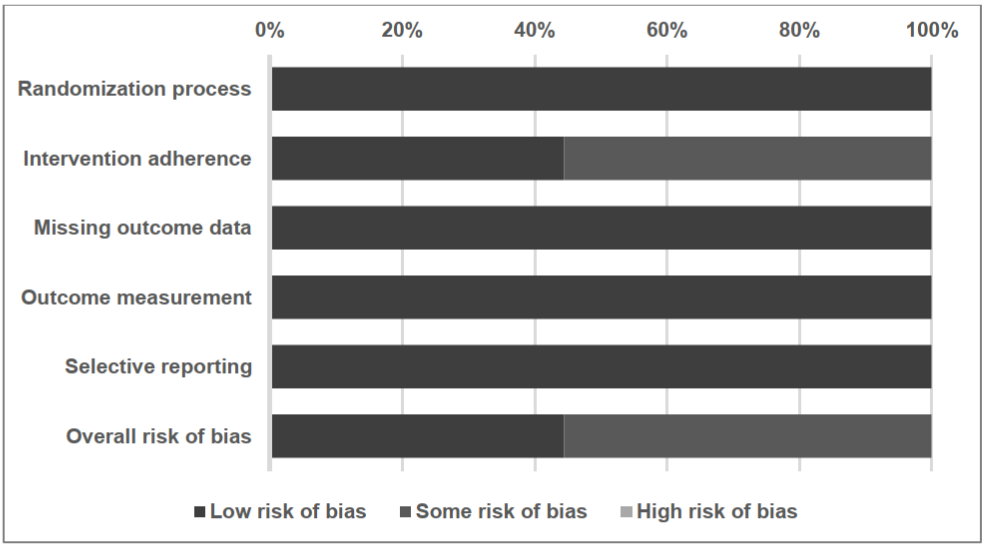

Figure S2 - The forest plot of pair-wise comparisons for different PPIs among post-PCI patients, retrieved from the included trials, demonstrates the risk ratio (RR) of MACEs.

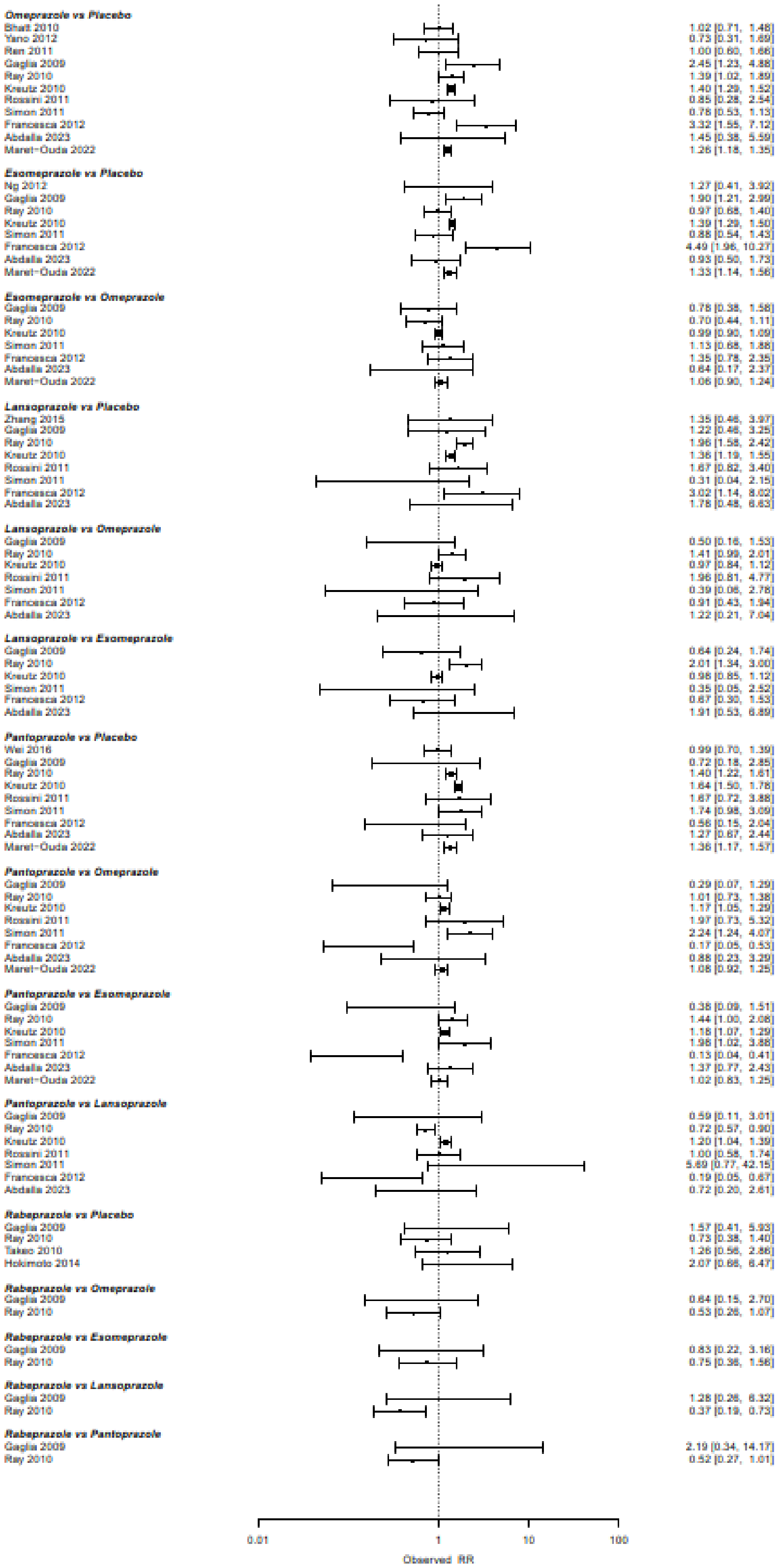

Figure S3 - Forest plots illustrating the risk ratio (RR) of individual outcomes of MACEs and mortality in clopidogrel concurrent with different PPIs and placebo groups among post-PCI patients. (a) Cardiac death. (b) Stroke. (c) Stent thrombosis. (d) Myocardial infarction. (e) Mortality.

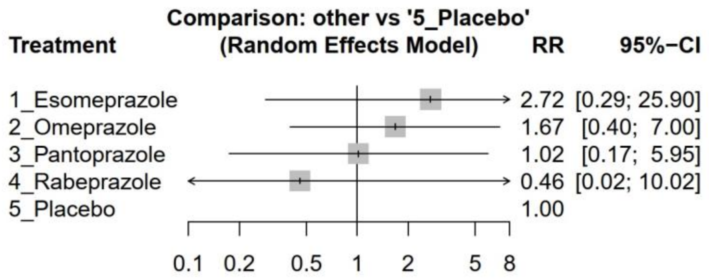

(a) Cardiac death

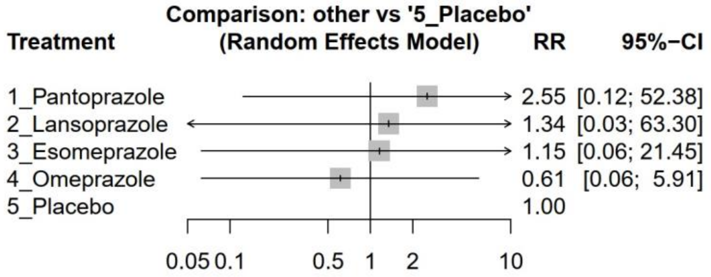

(b) Stroke

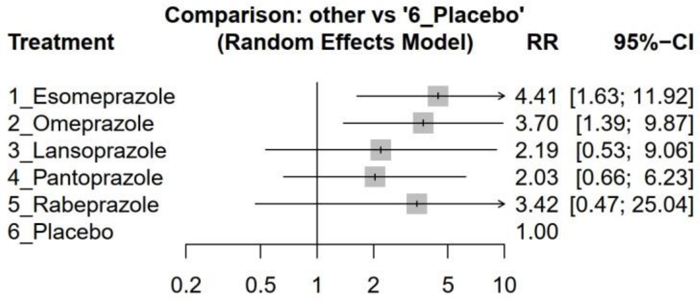

(c) Stent thrombosis

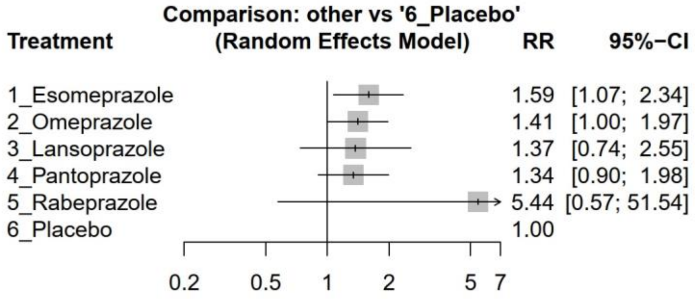

(d) Myocardial infarction

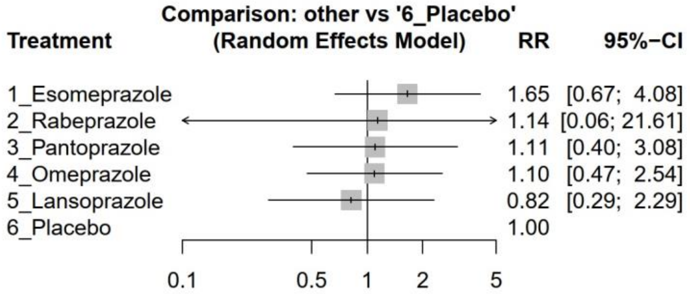

(e) Mortality

Figure S4 - The forest plot of pair-wise comparisons for different PPIs among post-PCI patients, retrieved from the included trials, demonstrates the risk ratio (RR) of GI bleeding.

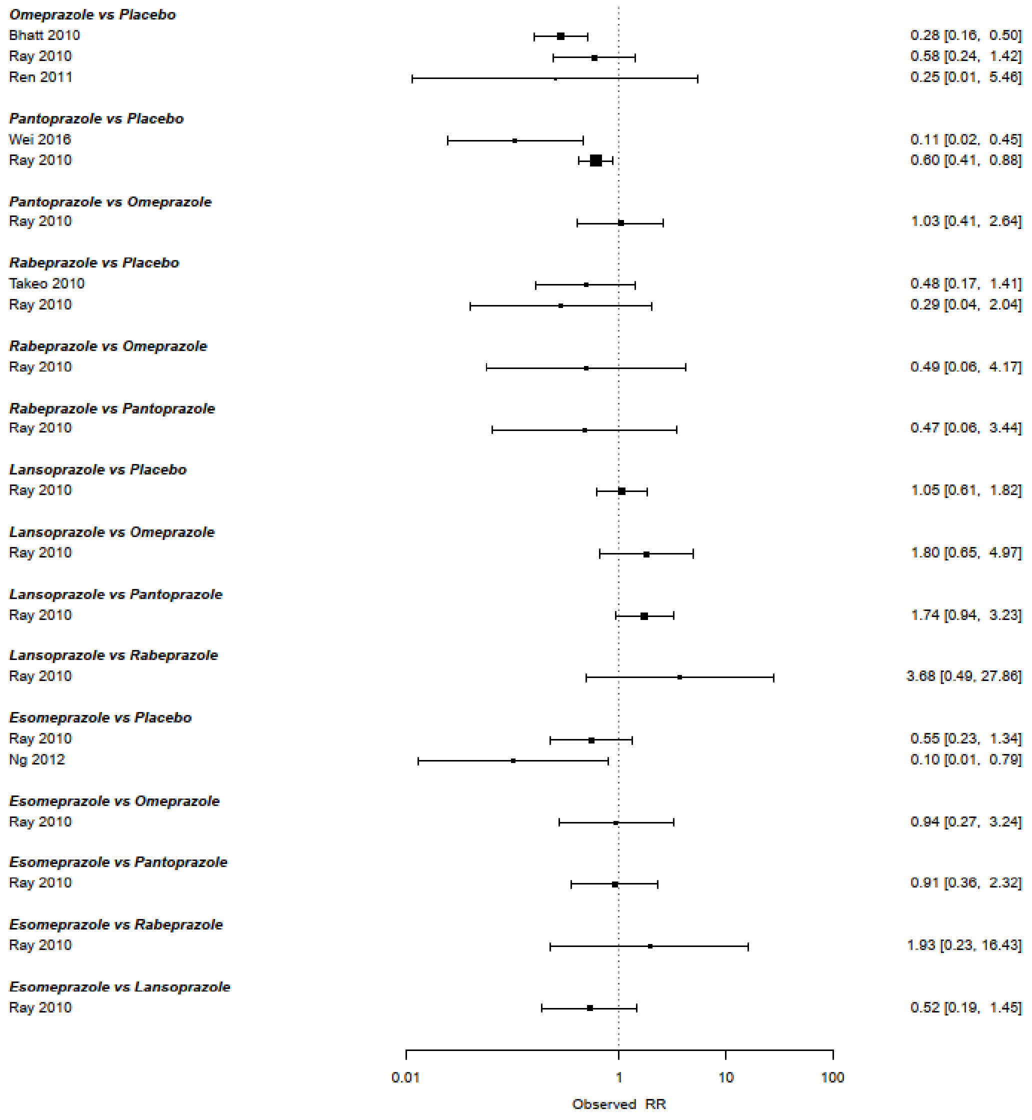

Figure S5 -Forest plots are presented for sensitivity analysis with the one-study removal method. Each of the 16 included studies was sequentially removed, with labels from a to p. Despite these removals, the rankings and clinical implications stayed consistent, suggesting that the inclusion or exclusion of any individual study does not modify the conclusions of our research.

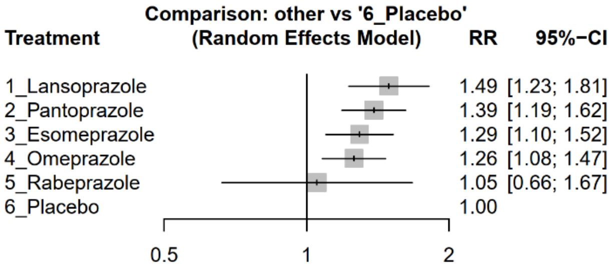

(a)

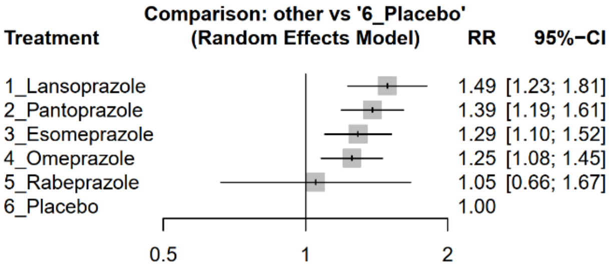

(b)

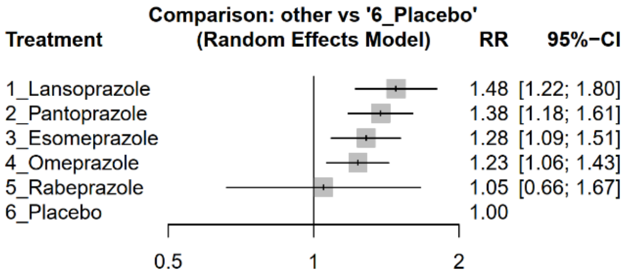

(c)

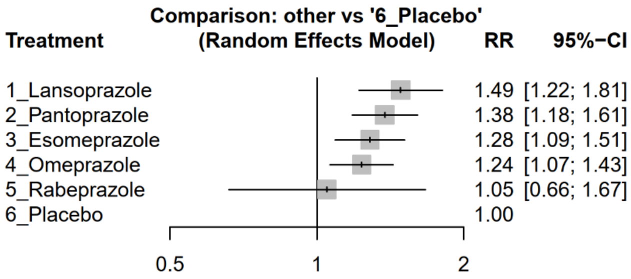

(d)

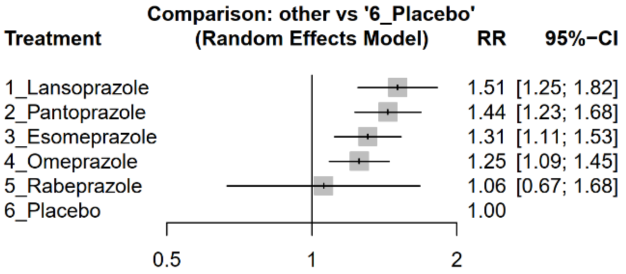

(e)

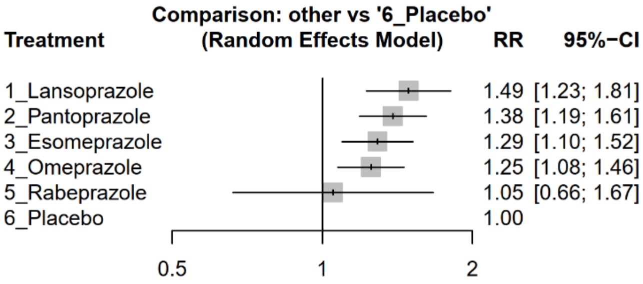

(f)

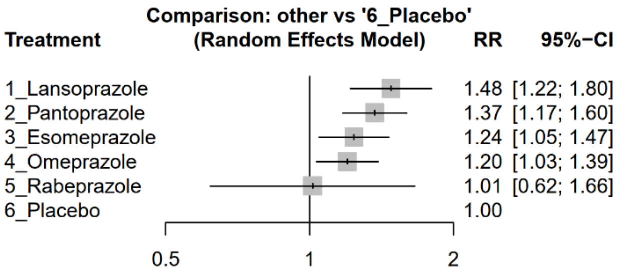

(g)

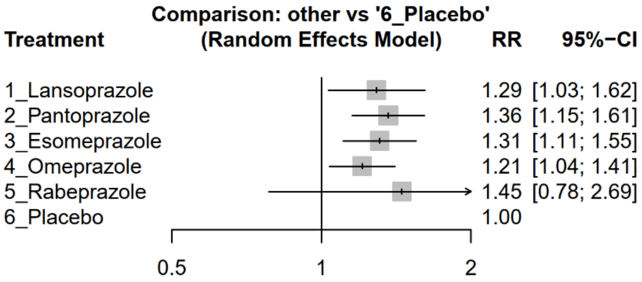

(h)

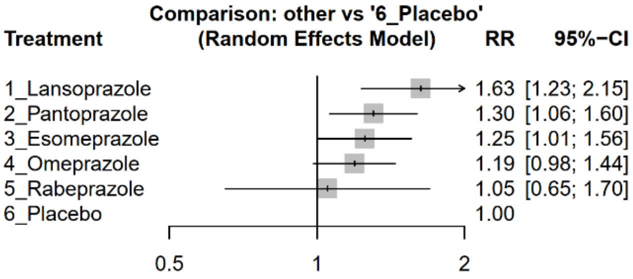

(i)

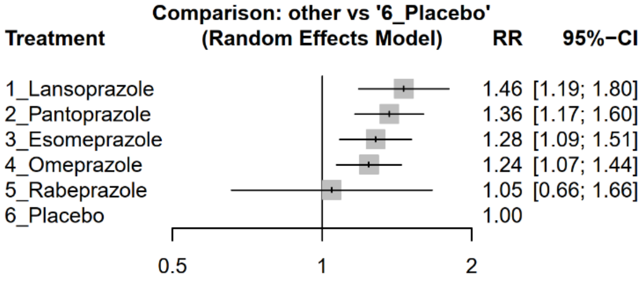

(j)

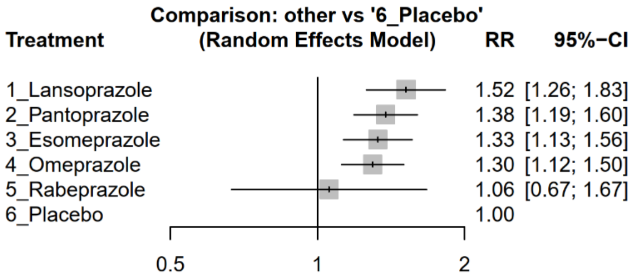

(k)

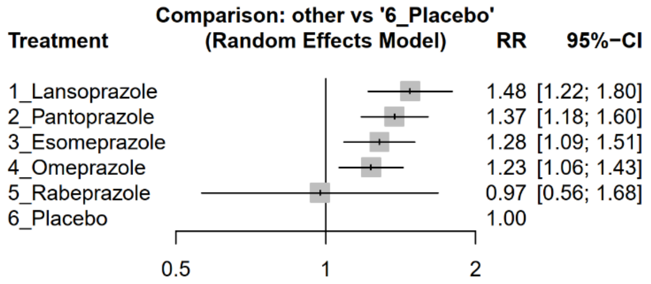

(l)

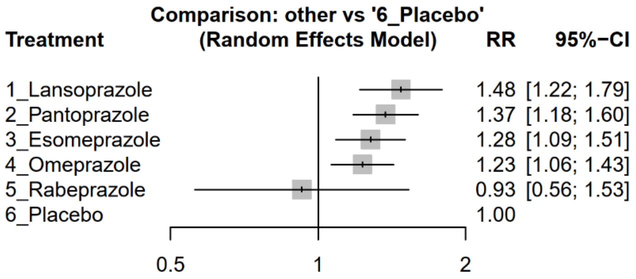

(m)

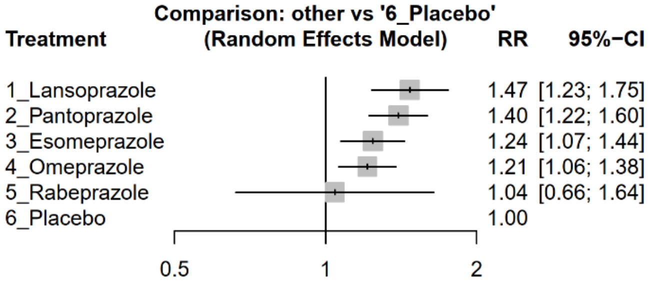

(n)

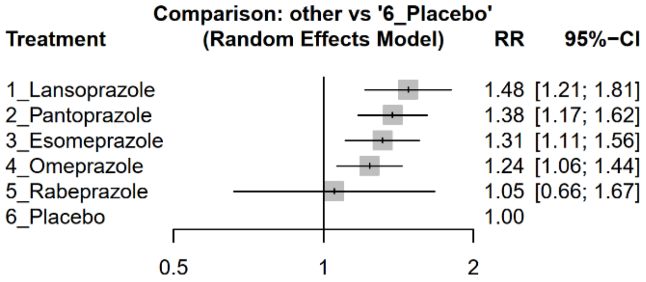

(o)

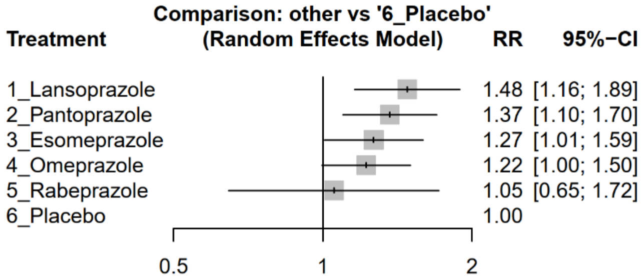

(p)

Figure S6 -Funnel plot for all paired comparisons that include the common comparator, which is the placebo group. The results of Egger's test produced a p-value of 0.302, suggesting an absence of significant publication bias.

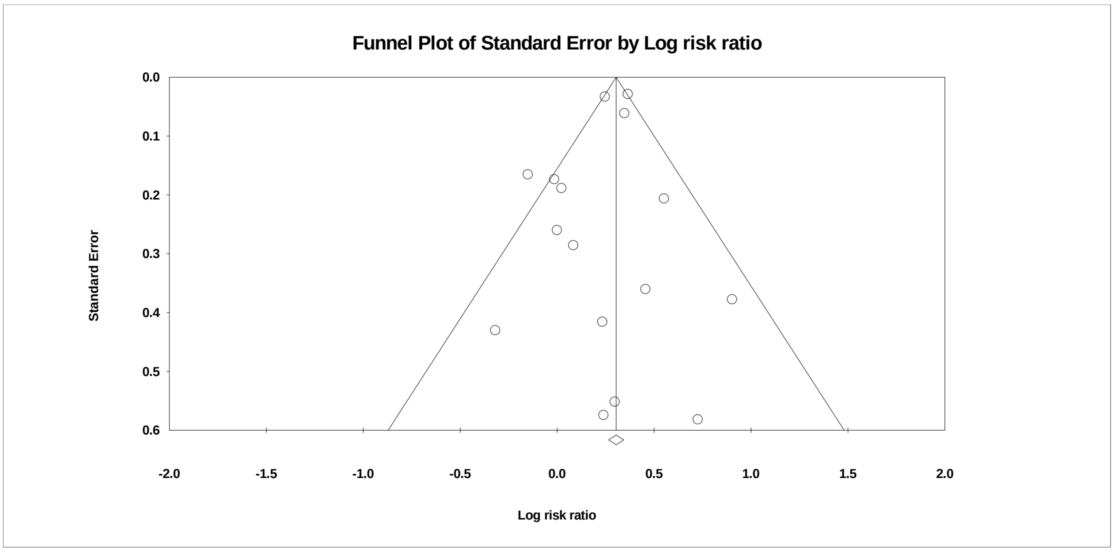

Supplement: Supplementary file 2 [file Table1.pdf]
